# Problematic Alcohol Use among University Students

**DOI:** 10.3389/fpsyt.2017.00086

**Published:** 2017-05-19

**Authors:** Tesfa Mekonen, Wubalem Fekadu, Tefera Chane, Shimelash Bitew

**Affiliations:** ^1^Psychiatry Department, College of Medicine and Health Sciences, Bahir Dar University, Bahir Dar, Ethiopia; ^2^College of Medicine and Health Sciences, Wollo University, Dessie, Ethiopia; ^3^School of Public Health, College of Health Sciences and Medicine, Wolaita Sodo University, Wolaita Sodo, Ethiopia

**Keywords:** alcohol abuse, academic achievement, factors in students’ alcohol misuse, peer pressure, social phobia

## Abstract

**Background:**

Alcohol is attributable to many diseases and injury-related health conditions, and it is the fifth leading risk factor of premature death globally. Hence, the objective of this study was to assess the proportion and associated factors of problematic alcohol use among University students.

**Material and methods:**

Cross-sectional study was conducted among 725 randomly selected University students from November to December 2015. Data were collected by self-administered questionnaire, and problematic alcohol use was assessed by Alcohol Use Disorder Identification Test. Chi-square test was used to show association of problematic use and each variable and major predicators was identified using logistic regression with 95% confidence interval (CI); and variables with *p*-value less than 0.05 were considered statistically significant.

**Results:**

About 83 (11.4%) of the samples were problematic alcohol users of which 6.8% had medium level problems and 4.6% had high level problems. Significantly associated variables with problematic alcohol use among students were presence of social phobia (AOR = 1.7, 95% CI: 1.0, 2.8), lifetime use of any substance (AOR = 6.9, 95% CI: 3.8, 12.7), higher score in students cumulative grade point average (AOR = 0.6, 95% CI: 0.4, 0.9), and having intimate friend who use alcohol (AOR = 2.2, 95% CI: 1.3, 3.8).

**Conclusion:**

Problematic alcohol use among university students was common and associated with social phobia, poor academic achievement, lifetime use of any substance, and peer pressure. Strong legislative control of alcohol in universities is important to reduce the burden of alcohol.

## Introduction

Alcohol is the third leading preventable risk factor for the global burden of disease and responsible for 3.3 million deaths (5.9% of all global deaths). In 2012, World Health Organization reported that 7.6 and 4% of deaths were attributable to alcohol among males and females, respectively. Alcohol contributes to over 200 diseases and injury-related health conditions, mostly alcohol dependence, liver cirrhosis, cancers, and injuries. Alcohol misuse is the fifth leading risk factor of premature death globally; among people between the ages of 15 and 49 years, it is the first leading cause (1, 2).

Alcohol misuse was also reported as a strong predictor of students’ mental health in which, it was attributable for increased depressive symptoms accompanied with drinking to cope (3, 4), attempted suicide and self-harm behaviors (5, 6), and aggressive behaviors (7). Alcohol use was also associated with the use of novel psychoactive substances, a new trend exacerbated by the presence of alcohol (8). Moreover, students with problematic alcohol use are less likely to seek professional help for their mental health problem (9). Problematic alcohol use contributes to a significant proportion of students’ engagement in risky sexual behavior (10), poorer executive functions (11), and poor academic achievement (12–14).

The prevalence of problematic alcohol use in university students was widely variable and generally higher in developed countries (15). Students’ alcohol use in general ranged from 22 in Ethiopia to 94% in Ireland; and problematic alcohol use ranged from 2.5 in Germany to 53% in Ireland (15–18). In developed countries, on average, each student had 1.7 drinks daily with about three episodes abusive drinking per month and about 10% of probable alcohol dependence (18, 19). Alcohol was also common in Ethiopian university students in which 31% of students in Addis Ababa University and about 50% in Haramaya University reported current alcohol use (20, 21). Students consumed alcohol for the reason of social gathering and as the means of social interaction influenced by their peers (16, 19, 22). Many predictors of problematic alcohol use have been identified previously. Sex, socioeconomic status, living in dormitory with higher number of roommates, cultural norms about alcohol, and having families who use substance were the commonly mentioned factors affecting students’ problematic alcohol use (5, 16, 23, 24).

Problematic alcohol use by university students is an important public health issue due to its multiple and wide range of effects on physical, psychosocial, and mental health. Despite some researchers tried to show the prevalence of alcohol use among university students in Ethiopia, there is little/no evidence on how many of those students engaged in problematic alcohol use and probable alcohol dependence. Therefore, it is very important to conduct surveys on students’ alcohol use problem by including further possible predictors. Hence, the purpose of this study was to assess the level of problematic alcohol use and predictor factors among university students.

## Materials and Methods

Cross-sectional study was conducted among 725 randomly selected Wolaita Sodo University regular students from November to December 2015. Wolaita Sodo University is one the government owned Universities in southern Ethiopia. Regular students enrolled in the University in 2015/16 academic year were included in the study.

Problematic alcohol use was the outcome variable; and the independent variables were sociodemographic variables (sex, age, marital status, religion, residence, monthly pocket money, and ethnicity), substance use related variables (having intimate friend who use alcohol, parents alcohol use status, and lifetime substance use), and other variables (students’ interest in their field of study, any disabling or chronic illness, parenting style, and academic performance).

Data were collected by pretested self-administered questionnaire by trained data collectors and supervisors. Problematic alcohol use was assessed by Alcohol Use Disorder Identification Test (AUDIT), which was developed by World Health Organization to be used in culturally diversified settings. AUDIT has 10 items in which the first 8 items are Likert scale scored from 0 to 4 and the last two items are scored as 0, 2, and 4. Total score of 8 or more was considered as positive for problematic alcohol use. AUDIT scores in the range of 8–15 represented low level of alcohol problems, whereas scores of 16 and above represented high level of alcohol problems; and AUDIT scores of 20 and above clearly showed alcohol dependence (25). Social phobia was assessed by using the Social Phobia Inventory (SPIN), which was developed by Connor et al. It was 17 items self-rating tool scored from 0 (not at all) to 4 (extremely) and the sum score ranged from 0 to 68 (26). A student with a score of 20 and above on SPIN were considered as having social phobia. The other variables were assessed by structured questionnaire developed by reviewing different literatures; and the responses were based on the students report.

Data were entered in to EPI info software version 3.5.3 and then transported to SPSS version 20 for analysis. Descriptive statistics were used to describe the result with respect to problem alcohol use with corresponding chi-square and *p*-value. Logistic regression was used to analyze the association between dependent and independent variables. Odds ratios with 95% confidence intervals (CIs) were used to show the strength of the associations, and variables with *p*-value of less than 0.05 were considered as statistically significant.

Ethical clearance was obtained from institutional review board of Wolaita Sodo University. The purpose of the study was fully explained for all participants in order to give them an informed choice to participate. Confidentiality was maintained by anonymous questionnaire and written informed consent was obtained from each participants.

## Results

### Sociodemographic Characteristics of Respondents

From the total of 740 sample size, 725 participants were included in the study with the response rate of 97.97%. The mean age of the participants was 21.15 years (SD ± 1.7) and the reported monthly pocket money of the students ranged from 50 to 3,600 Ethiopian birr with the median value of 300 Ethiopian birr (Table 1).

**Table 1:** **Sociodemographic characteristics of the participants with respect to problematic alcohol use (*n* = 725)**.

Variables		Problematic alcohol use		χ^2^	df	*p*-Value
		No, *n* (%)	Yes, *n* (%)			
Sex	Male	425 (58.6)	57 (7.9)	0.20	1	0.65
	Female	217 (29.9)	26 (3.6)			
Marital status	Single	527 (72.7)	58 (8)	7.62	2	0.02
	In open relationship	80 (11)	19 (2.6)			
	Others^a^	35 (4.8)	6 (0.8)			
Residence	Urban	268 (37)	45 (6.2)	4.70	1	0.03
	Rural	374 (51.6)	38 (5.2)			
Religion	Orthodox	253 (34.9)	38 (5.2)	4.30	2	0.23
	Protestant	300 (41.4)	40 (5.5)			
	Muslim	74 (10.2)	4 (0.6)			
	Others	15 (2.1)	1 (0.1)			
Year of study	1st year	176 (24.3)	16 (2.2)	6.54	4	0.16
	2nd year	198 (27.3)	23 (3.2)			
	3rd year	139 (19.2)	27 (3.7)			
	4th year	74 (10.2)	8 (1.1)			
	5th year	55 (7.6)	9 (1.2)			
Parenting style	Democratic	516 (71.2)	59 (8.1)	3.86	1	0.04
	Restrictive	126 (17.4)	24 (3.3)			

*^a^Married, divorced, and widowed*.

### Substance Use and Related Characteristics of the Participants

From the total participants, 160 students had parents with alcohol use history and 29 (4%) of them were screened positive for alcohol use problem. Lifetime substance use (alcohol, tobacco products, or khat) was also reported by 240 students (Table 2).

**Table 2:** **Substance use and related characteristics of the participants with respect to problematic alcohol use (*n* = 725)**.

Variables		Problematic alcohol use		χ^2^	df	*p*-Value
		No, *n* (%)	Yes, *n* (%)			
Parents alcohol use history	Yes	131 (18.1)	29 (4.0)	9.03	1	0.003
	No	511 (70.5)	54 (7.4)			
Alcohol user intimate friend	Yes	185 (25.5)	53 (7.3)	40.9	1	<0.0001
	No	457 (63.0)	30 (4.1)			
Lifetime substance use^a^	Yes	174 (24.0)	66 (9.1)	91.18	1	<0.0001
	No	468 (64.6)	17 (2.3)			
Interest in their department	Yes	547 (75.4)	70 (9.7)	0.04	1	0.84
	No	95 (13.1)	13 (1.8)			
Probable social phobia	No	356 (49.1)	33 (4.6)	7.28	1	0.007
	Yes	286 (39.4)	50 (6.9)			

*^a^Use of substances (alcohol, khat, and tobacco products) in their lifetime*.

### Prevalence and Associated Factors of Problematic Alcohol Use

The overall prevalence of problematic alcohol use among university students was 11.4% (95% CI: 9.1, 13.7) with 6.8% medium level problematic alcohol use and 4.6% high level problematic alcohol use. The occurrence of problematic alcohol use in male to female ratio was about 2:1. Variables that were significantly associated with problematic alcohol use of students were having alcohol user intimate friend (AOR = 2.2, 95% CI: 1.3, 3.8), ever use of substance (AOR = 6.9, 95% CI: 3.8, 12.7), academic achievement (grade point) (AOR = 0.6, 95% CI: 0.4, 0.9), and probable social phobia (AOR = 1.7, 95% CI: 1.0, 2.8) (Table 3).

**Table 3:** **Predictors of alcohol use problem among university students (*n* = 725)**.

Variables		Problematic alcohol use		COR (95% CI)	AOR (95% CI)
		Yes	No		
Marital status	Single	58	527	1	1
	In relation ship	19	80	2.2 (1.2–3.8)	1.6 (0.8–3.1)
	Others^a^	6	35	1.6 (0.6–3.9)	1.5 (0.5–4.3)
Residence	Urban	45	268	1.7 (1.0–2.6)	1.3 (0.8–2.1)
	Rural	38	374	1	1
Alcohol user close friend	Yes	53	185	4.4 (2.7–7.0)	2.2 (1.3–3.8)**
	No	30	457	1	1
Parents alcohol use	Yes	29	131	2.1 (1.3–3.4)	0.7 (0.4–1.3)
	No	54	511	1	1
Parenting style	Democratic	59	516	0.6 (0.4–1.0)	0.8 (0.4, 1.5)
	Restrictive	24	126	1	1
Lifetime substance use	Yes	66	174	10.4 (5.9–18.3)	6.9 (3.8–12.7)*
	No	17	468	1	1
Academic performance				0.4 (0.2–0.6)	0.6 (0.4–0.9)
Probable social phobia	No	33	356	1	1
	Yes	50	286	1.9 (1.2–3.0)	1.7 (1.0–2.8)

*^a^Married, divorced, and widowed*.

*p-value less than 0.001.

**p-value less than 0.01.

## Discussion

Approximately 1 from 10 students had problematic alcohol use and 4.6% students were in high level problematic alcohol use, which was indicative for alcohol dependence. The finding was consistent with the Belgian University students in which problematic alcohol use was 14 and 3.6% probable dependence (14). It was also in line with the findings of international study for Bulgaria 9%, France 8.5%, and Hungary 8% (15). However, our finding was generally lower as compared with many other countries’ problematic alcohol use among college students. For instance, 47.6% of students in Spain were binge drinkers (27), 61% of students in England were positive for AUDIT scores (18), 40–45% of American college students were heavy drinkers (28), and South Africa 15% (15). The current finding was also much lower than the findings from Belgium 36.5%, Colombia 35%, Ireland 53%, and the study done in Italian young people in which binge drinking was reported by 67.6% of the participants (15, 29).

The main possible explanation for this difference might be the difference in socioeconomic status of the students. It was suggested by researchers that high socioeconomic status might increase the risk of problem alcohol use (15). Race was also another important explanation for the above discrepancy; the percentage of students who reported hazardous drinking was five times higher in whites than non-whites (18, 22, 28). Use of self-administered survey in the current study might also underestimate the prevalence of problematic alcohol use. On the other hand, the current finding was higher as compared with findings from Germany, Greece, Romania, and Italy in which students’ problem use of alcohol were 2.5–4.5% in those countries (15).

Peer pressure was an important predictor of problematic alcohol use in university students in which the odds of having problematic alcohol use among students who had alcohol user intimate friends were more than two times as compared with their counter parts. Direct or indirect encouragement from intimate friends was indicated as a major factor to be engaged in risk-taking behaviors. Students tend to drink more alcoholic beverages during social gatherings in the virtue of social interaction and high level of alcohol abuse was reported by students living in the dormitory with high density of roommates (19, 22, 30). In a Kenyan study, 75.1% of students admitted that they were introduced for substance abuse by their friends. Previous studies in Ethiopia also showed the influence of peers on students’ alcohol consumption; students whose friends consume alcohol were more likely to consume alcohol (16, 31).

Lifetime exposure for substances other than alcohol, like Khat and tobacco products was strongly and positively associated with students’ problematic alcohol use. Students who had ever used any substance other than alcohol were about seven times more likely to engage themselves in problematic alcohol use. This was supported by the study conducted in Ethiopia among medical students by which ever use of cigarette or khat was associated with current alcohol use (16). Having history of smoking was associated with higher risk of alcohol use disorder than who never smoked as implicated in previous studies (32). Individuals who used some kind of substance also engaged in further risk-taking behaviors like alcohol abuse and subsequent alcohol use disorder (10).

This research showed that high achievement in students’ academic performance was associated with low risk of problematic alcohol use. Alcohol abuse has significant effect on executive functions of an individual accompanied with blackouts, frequent injuries, less motivation in activities, and even moderate doses of alcohol use had risk of decreasing academic performance (11, 33). Even though there were some differences in male and female students (34), this finding was consistent with other studies conducted in different areas (13, 14, 35).

The other variable significantly associated with students problematic alcohol use in the current study was probable social phobia. Students who were screened positive for probable social phobia were 1.7 times more likely to have problematic alcohol use and this was supported by other studies in Nigeria and USA (36). This might be explained by the students’ tendency to use alcohol as self-medication to get away from their stress and fear (37–39).

Unlike other studies (16, 24, 31), sex, marital status, family history of substance use, and student’s origin of residence were not associated with problematic alcohol use. Even though the occurrence of problematic alcohol use in male to female ratio was 2:1, it was statistically not significant in the current finding.

### Limitations of the Study

This study did not assess all possible associated substances and did not show the interaction effect of other substance use with problematic alcohol use. Since alcohol is a gateway drug, it is expected that students would also use other drugs in addition to alcohol. Even though we had tried to maintain privacy and encourage the participants to complete the real information, the result might be underestimated due to social desirability bias. The study also did not measure blood alcohol concentration of the participants, which would be very important to assess the current level of alcohol in the blood stream.

## Conclusion

Significant proportion of students reported problematic alcohol use and was associated with peer pressure, lifetime substance use, poor academic performance, and probable social phobia. Developing preventive interventions focusing on risky behaviors and legislative control in the universities toward alcohol use problem is important. Further research is needed to indicate the combined effect of other substances to problematic alcohol use, and it would be also very important to assess depressive symptoms associated with alcohol.

## Ethics Statement

Ethical clearance was obtained from institutional review board of Wolaita Sodo University. The purpose of the study was fully explained for all participants in order to give them an informed choice to participate. Confidentiality was maintained by anonymous questionnaire and written informed consent was obtained from each participants.

## Author Contributions

All authors contributed equally for the whole process of this study. All authors read and approved the final manuscript.

## Conflict of Interest Statement

The authors declare that the research was conducted in the absence of any commercial or financial relationships that could be construed as a potential conflict of interest.
